# Internet‐based third‐wave Cognitive Behavioral Therapy (CBT) for reducing stress in parents of children and adolescents with chronic conditions: Systematic review and meta‐analysis protocol

**DOI:** 10.1002/hsr2.70125

**Published:** 2024-10-06

**Authors:** Maria Davey, Leanne Fried, Hui Jun Chih, Rosanna Rooney, Alison Roberts

**Affiliations:** ^1^ Curtin School of Population Health Curtin University Kent Street Bentley WA Australia; ^2^ Telethon Kids Institute Perth Children's Hospital 15 Hospital Avenue Nedlands WA Australia; ^3^ Child and Adolescent Health Service Perth Children's Hospital 15 Hospital Avenue Nedlands WA Australia

**Keywords:** ‘child’, ‘chronic condition’, ‘online intervention’, ‘parent’, ‘stress’, ‘third generation’

## Abstract

**Background:**

Parents of children and adolescents with chronic conditions have an increased risk of stress‐related mental health problems, and reduced quality of life. Third wave Cognitive Behavioral Therapy (CBT) interventions have been shown to reduce stress in this parent population. Studies demonstrate that this efficacy endures when these therapies are delivered online. The aim of this protocol is to describe the methodology and methods that will be employed for a systematic review and meta‐analysis that investigates the effectiveness of internet‐based third‐wave CBT interventions for parents of children and adolescents with chronic conditions, and their potential to reduce stress for parents.

**Methods/Design:**

This systematic review will follow the Preferred Reporting Items for Systematic Review and Meta‐Analyses (PRISMA) approach. A search of peer‐reviewed journal articles published from January 1970 to December 2022 will be undertaken in the following databases: CINHAHL, EMBASE, EMCARE, MEDLINE, PsycINFO. Title and abstract screening together with data extraction will be completed by two reviewers, and will be arbitrated by a third reviewer, should there be any discrepancies. The risk of bias will be assessed using the Cochrane Risk of Bias tool. Data related to the primary outcome (i.e. reduction of stress in parents) will be extracted for analysis.

**Results:**

This Systematic Review and Meta‐Analysis plans to provide a conclusive overview of the available evidence on the effectiveness of internet‐based third‐wave parent interventions and their ability to reduce stress in parents of children and adolescents with chronic conditions. If the results of this analysis prove positive, further research can be undertaken to support this vulnerable parent population. The findings of the review will be published in a peer‐reviewed journal.

**Discussion:**

Third‐wave internet‐based approaches may show great promise in supporting parents to cope with the stress/distress associated with parenting a child with a chronic condition. This protocol will guide a systematic literature review of the evidence for internet‐based third‐wave interventions for this parent population.

**Registration:**

This systematic review was registered on PROSPERO on 24th June, 2022 (Registration: CRD42022337334).

## 1 BACKGROUND

Although the prevalence of childhood chronic conditions has doubled over the last 20 years,^1^, ^2^ many children and adolescents with chronic conditions are living longer due to better management and increased use of medical technologies for chronic conditions.^3^ A chronic medical condition is a condition that affects daily life, requires ongoing management and treatment, and needs regular medical attendance.^4^ Common chronic conditions that present in children and adolescents include asthma, diabetes, epilepsy/seizures, obesity, cystic fibrosis, allergies, sickle cell anemia, irritable bowel diseases (e.g. ulcerative colitis, Crohn's disease), migraines/headaches, juvenile arthritis, congenital heart defect, traumatic brain surgery/spinal injury, and organ transplant.^5^ Over time there has been growing interest amongst health professionals in how, and to what extent, childhood chronic conditions affect other family members, especially parents.^6^

Parents of children with chronic conditions often face high stress and burnout,^7^ especially before diagnosis, at diagnosis, and during adjustment to managing the condition.^8^ Parenting stress is a psychological reaction that may be viewed as a negative response when parenting demands are inconsistent with parents’ expectations, and/or when parents do not have the resources to meet the demands.^9^ Parenting stress has been shown to minimize the ability of parents to effectively manage their child's chronic condition (e.g., reduced parental vigilance resulting in poor adherence to disease management regimes).^10^ Chronic conditions that require ongoing management, have an uncertain prognosis, and require a high level of care (e.g. Type 1 Diabetes), may be particularly impacted by parenting stress. Increased parenting stress has been associated with poor psychological adjustment for the parent and the child,^11^ together with negative child behavior (i.e. externalizing problems), and a lack of positive child outcomes (i.e. inability to manage affective, social and achievement challenges).^12^ Research shows that these parents often struggle with caregiving, lack confidence in managing their child's condition, and face challenges in ensuring their child's optimal well‐being.^13^ Parents also experience increased anxiety and depression symptoms, added financial strain, and an increase in family conflict.^7^, ^14^, ^15^ Increased anxiety and depression symptoms may negatively impact not only parenting practices, but also family functioning.^16^, ^17^

The above research supports the negative effects of parenting stress on many parent and child outcomes. Given that having a child with a chronic condition increases parenting stress,^18^ it is important to explore interventions that provide parents with skills and strategies to better manage the stress they experience, due to the challenges and hardships they face. Parent‐based cognitive behavior therapy (CBT) interventions have been found to reduce children's primary symptoms,^6^ together with easing parental distress when managing a child with a chronic condition.^19^

Over the last 20 years there has been increased interest in the third wave CBT intervention approach.^20^ The third wave approach provides more flexible and holistic ways to enhance mental well‐being by incorporating components such as acceptance, mindfulness and commitment to values‐based living.^21^, ^22^ Research has suggested that the third wave approach be extended to parents by enhancing current behavioral parenting interventions with mindfulness to investigate the effects of the mindfulness‐based intervention approach on parenting and parental adjustment.^23^, ^24^

The main aims of the third wave approach are to promote a mindful relationship with both thoughts and emotions, promote acceptance of these thoughts and emotions, and build life coping skills according to one's chosen values.^25^, ^26^ These approaches include, but are not limited to, Acceptance and Commitment Therapy (ACT),^27^ Behavior Activation (BA),^28^ Cognitive Behavioral Analysis System of Psychotherapy (CBASP),^29^ Compassion Focused Therapy/Compassionate Mind Training (CT/CMT),^30^ Functional Analytic Psychotherapy (FAP),^31^ Mindfulness Based Cognitive Therapy/Mindfulness Based Stress Reduction (MBCT/MBSR),^32^ and Dialectical Behavior Therapy (DBT).^33^

Research has reported a link between lower parenting stress and higher parental mindfulness.^34^, ^35^ Accordingly, a growing number of studies have delivered mindfulness‐based interventions to parents, with the aim of reducing parenting stress and improving psychological outcomes for youth.^36^, ^37^ Self‐compassion has been identified as an equally or more effective target of intervention in improving parent and child outcomes.^38^ This may be due it it being a narrower concept than general mindfulness as its reference is to negative thoughts and feelings related to experiences solely of personal suffering, rather than all experiences.^39^ Self‐compassion may be an effective target for increasing parenting quality and decreasing parental distress.^38^, ^40^, ^41^

Although research has explored traditional face‐to‐face interventions for this parent population, more research is needed on alternative ways to support parents, considering the many challenges they face (i.e. limited access to services, time constraints, lack of self‐care).^42^ It has been suggested that parents of children with chronic conditions may benefit from more novel models of delivering support, such as internet‐based interventions.^43^ The advantages of internet‐based interventions include instant access, regularly updated content, easy parent engagement for support and answers, and the ability to track parents’ progress.^44^, ^45^ The internet is also a promising delivery mode as interventions can be scaled, are cost‐effective,^46^ and have the potential of reducing the barriers of current face to face interventions due to their flexibility and accessibility.^47^

Research has reported that internet‐based parenting interventions are effective in increasing positive parenting behaviors and reducing parent stress (i.e. small effect).^48^ A recent Cochrane review on psychological interventions for parents of children and adolescents with chronic illness,^49^ examined 44 randomized controlled trials and identified eight studies that were delivered via the internet. These studies included five CBT studies^19^, ^50^, ^51^, ^52^, ^53^; two Problem Solving Therapy (PST) studies^54^, ^55^; and one Motivational Interviewing (MI) study.^56^ This review provides evidence‐based strategies for managing parent challenges and enhancing psychosocial adaptation for both the parent and the child. Although this review is an important addition to the literature, evidence around third‐wave interventions is unknown, despite internet‐based parenting interventions being. the most used form of delivering technology‐based parenting interventions.^57^, ^58^ Research is needed on internet‐based third wave interventions to help parents of children with chronic conditions manage parenting stress using evidence‐based strategies. The International Prospective Register of Systematic Reviews (PROSPERO) was searched and to date there is no systematic review or meta‐analysis published that investigates the effectiveness of internet‐based third wave CBT interventions for this parent population, and their potential in reducing parent stress. This highlights a key gap, which will be addressed through a systematic review and meta‐analysis of randomized controlled trials to assess the effectiveness of internet‐based third wave interventions in reducing stress in parents of children and adolescents with chronic conditions.

## 2 PRIMARY AIM

The primary aim of this research is to conduct a systematic review and meta‐analysis to explore the effectiveness of internet‐based third wave interventions on reducing stress in parents of children and adolescents with chronic conditions.

### 2.1 Primary review question

Are internet‐based third wave CBT interventions, when compared to an active control or wait‐list control, more effective in reducing stress in parents of children and adolescents with chronic conditions?

### 2.2 Secondary review questions

a) Does the type of chronic condition moderate the effectiveness of the internet‐based third‐wave CBT intervention? (e.g. Type l Diabetes vs. Asthma)
b) Does the delivery mode moderate the effectiveness of the internet‐based third‐wave CBT intervention? (e.g. app on phone vs. intervention via video conferencing)

## 3 METHODOLOGY

This systematic review will follow the procedures outlined in the Cochrane Handbook for Systematic Reviews of Interventions,^59^ and the PRISMA‐P (Preferred Reporting Items for Systematic Review and Meta‐Analysis Protocols),^60^ as outlined below.

### 3.1 Data sources and search strategy

The following databases will be systematically searched, using the advanced search function where possible; MEDLINE via Ovid, Excerpta Medica database (EMBASE) via Ovid, EMCARE via Ovid, Cumulative Index to Nursing and Allied Health Literature (CINAHL) via EBSCO, PsycINFO via Ovid. Medical subject heading (MeSH) terms and text words will be adopted, mainly including “cognitive behavioral therapy”, “internet‐based intervention”, and “chronic disease”. Keywords “online intervention”, “third generation”, “parent”, “stress”, “child”, “chronic condition” will be used in the search. The details of the search terms using truncation and wildcards when available to accommodate for different spellings, can be found in Appendix A. The number of records located in each database will be noted in the format of the PRISMA flow diagram. Full details of the articles identified will be imported to an EndNote library. Any duplicates of identical records will be removed, and the total number of duplicates recorded in the PRISMA flow diagram.

### 3.2 Criteria for study selection

Titles and abstracts of retrieved studies will be screened independently by two reviewers of the research team (MD & LF) to identify studies that potentially meet the inclusion/exclusion criteria. The inclusion criteria for this review are experimental studies of third‐wave CBT interventions: Acceptance and Commitment Therapy (ACT),^27^ Behavior Activation (BA),^28^ Cognitive Behavioral Analysis System of Psychotherapy (CBASP),^29^ Compassion Focused Therapy/Compassionate Mind Training (CT/CMT),^30^ Functional Analytic Psychotherapy (FAP),^31^ Mindfulness Based Cognitive Therapy/Mindfulness Based Stress Reduction (MBCT/MBSR)^32^ and Dialectical Behavior Therapy (DBT),^33^ with an adequate control group, published in peer‐reviewed journals; experimental studies that reported the baseline and follow‐up stress/mental wellbeing of the parents of children and adolescents with chronic conditions at least four weeks after the intervention. All peer‐reviewed studies published in English between January 1970 and December 2022 that met the inclusion criteria will be included in the review. The exclusion criteria are studies that reported outcomes immediately following the intervention without a follow‐up at least four weeks post‐intervention. Any selection disagreements will be resolved by a third reviewer of the research team (HJC).

All studies will be screened and selected based on the PICOR format as follows:

#### 3.2.1 P(opulation)

The population of interest of all eligible studies will be parents of children and adolescents with chronic conditions. Children and adolescents with any chronic condition will be included in this study.

#### 3.2.2 I(ntervention)

Internet‐based third wave CBT interventions will be included. Third wave CBT interventions are defined as a collection of interventions that target the process of thoughts, rather than their content, to help individuals become aware of their thoughts and accept them in a non‐judgemental way.^61^ The interventions include ACT, BA, CBASP, CT/CMT, FAP, MBCT/MBSR, DBT as outlined earlier in this paper.

#### 3.2.3 C(omparator)

Comparable control group, including wait‐list control group.

#### 3.2.4 O(utcome)

The primary outcome is a reduction in stress in parents of children and adolescents with chronic conditions from baseline to at least four weeks post intervention, where the intervention is an internet‐based third‐wave CBT intervention. This outcome will be assessed via any valid and reliable scale that measures stress pathology and may include, but is not limited to: The Parental Stress Scale^1^ (PSS^1^), The Perceived Stress Scale^2^ (PSS^2^) The Holmes and Rahe Stress Scale; The Standard Stress Scale (SSS); the Depression, Anxiety and Stress Scale (DASS‐21/42). Additional outcomes include any reported harms/adverse effects of the internet‐based third wave CBT interventions.

#### 3.2.5 R(esearch design)

Experimental studies, including randomized controlled trials, and experimental/quasi‐experimental studies will be included in this study. Full texts of these potentially eligible studies will be retrieved and independently assessed by two reviewers of the research team (MD & LF). Disagreements will be resolved by a third reviewer of the research team (HJC).

### 3.3 Data extraction

A standardized form will be used to extract data from the included studies. For each study, the specific extracted information will be noted in a table and will include: 1) article information (authors, countries, publication years, funding source), 2) study characteristics (aims of study, study design, study duration, recruitment criteria, sampling technique, unit of randomization), 3) participant's characteristics (age gender, chronic condition of the adolescent, etc.), 4) intervention group details (mode of delivery, session topics, length, frequency, duration, etc. including those of the comparable control group, 5) outcomes (stress, number lost to follow‐up, means, SD and effect sizes, etc.). Should the data of key outcomes not be listed, the authors of the selected articles will be contacted to obtain

this information. The data from all selected articles will be extracted by one independent reviewer (MD). The data will be verified by a second reviewer (HJC).

### 3.4 Assessment of risk of bias

The quality of the included studies will be assessed following the Cochrane Collaboration's

tool for assessing the risk of bias.^59^ The risk of bias will be assessed according to the following five domains: a) bias arising from the randomization process; b) bias due to deviations from intended interventions; c) bias due to missing outcome data; d) bias in the measurement of the outcome; and e) bias in the selection of the reported results. Risk of bias judgment for each domain and an overall judgment will be made in terms of *low risk of bias, some concerns*, or *high risk of bias*. Two reviewers of the research term (MD & LF) will independently perform the assessment, and a third reviewer (HJC) will be responsible to resolve any discrepancies until a consensus is reached.

### 3.5 Data synthesis and analysis

Following data extraction, a narrative synthesis of the outcomes of the selected studies will be presented in the final review. This synthesis will report the characteristics of the included studies, including general information, settings, participants, number of chronic conditions, interventions, controls/comparators, outcomes, and number of follow‐ups. Key information of the study intervention will be summarized in terms of the theoretical and conceptual frameworks, specific content, duration of each session, session pattern, session frequency, and modes of delivery (online or online plus others). In doing this it is expected that the main features of the included studies can be clearly depicted.

Meta‐analyses will be conducted if at least three randomized controlled trials for a specific comparison are available. Heterogeneity of the shortlisted randomized controlled trials will

be assessed by inspecting the forest plot, I^2^ statistic, and τ^2^.^60^ A random effect model will be used to accommodate the heterogeneity. Effect size will be reported.

### 3.6 Analysis of Subgroups/Subsets

Types of chronic conditions and delivery modes will be analyzed to answer the secondary questions. The impact of the interventions on parents of children and adolescents with two or more chronic conditions will be analyzed separately from the parents of children and adolescents with a single chronic condition. Given the impossibility of providing a priori an exhaustive list of approaches, should other third‐wave CBT interventions be discovered, these will be reported as a subgroup in the systematic review.

### 3.7 Ethics and dissemination

The extracted data will be in aggregated form and there will be no access to the individual data being identified. Ethics approval is not required. This protocol has been registered on PROSPERO (Prospective Register of Systematic Review, registration number: CRD42022337334). The findings will be shared via PROSPERO and a peer‐reviewed journal.

## 4 RESULTS

The search terms are currently being piloted and finalized. Following selection and assessment of the publications, data extraction and analyses will begin. Data synthesis and presentation of the findings is anticipated to be completed by early 2024.

## 5 DISCUSSION

Parents of children and adolescents with chronic conditions report higher levels of parenting stress.^12^ Third‐wave CBT approaches show great promise in supporting parents to cope with the stress/distress associated with parenting a child with a chronic condition. Despite the convenience of parents’ preference towards internet‐based interventions, no systematic literature review and meta‐analyses have been performed to evaluate the effectiveness of internet‐based interventions in reducing parents’ stress. This protocol will guide a thorough evaluation of the published studies to assess the effectiveness of internet‐based third‐wave CBT approaches, and to identify characteristics of interventions that provide evidence of their effectiveness in reducing stress in parents of children and adolescents with chronic conditions.

## AUTHOR CONTRIBUTIONS

**Maria Davey**: Conceptualization; Investigation; Writing—original draft; Methodology; Validation; Writing—review and editing; Formal analysis; Data curation. **Leanne Fried**: Conceptualization; Supervision. **Hui Jun Chih**: Methodology; Supervision. **Rosanna Rooney**: Supervision. **Alison Roberts**: Supervision.

## CONFLICT OF INTEREST STATEMENT

The authors declare no conflict of interest.

## ETHICS APPROVAL

As this is a systematic review proposal of the existing literature, no ethics approval is required.

## TRANSPARENCY STATEMENT

The lead author Maria Davey affirms that this manuscript is an honest, accurate, and transparent account of the study being reported; that no important aspects of the study have been omitted; and that any discrepancies from the study as planned (and, if relevant, registered) have been explained.

## Data Availability

Data sharing not applicable to this article as no datasets were generated or analyzed during the current study.

## APPENDIX A ‐ Search Strategy

1 (‘online intervention*‘ or ‘web based intervention*‘ or ‘digital* based intervention*‘ or ‘tele* intervention*‘ or ‘e* intervention*‘ or ‘internet based intervention*‘ or ‘technology based intervention*‘ or ‘social media intervention*‘ or ‘app intervention*‘ or ‘cognitive behavio* therap*‘ or CBT or ‘cognitive behavio* analysis system of psychotherap*‘ or CBASP or ACT or ‘acceptance and commitment therap*‘ or ‘mindfulness based’ or MBCT or MBSR or ‘functional analytic psychotherap*‘ or FAP or ‘dialectical behavio* therap*‘ or DBT or ‘behavio* activation’ or BA or ‘compassion focused therap*‘ or CFT or ‘compassionate mind training’ or CMT). mp. [mp=title, abstract, original title, name of substance word, subject heading word, floating sub‐heading word, keyword heading word, organism supplementary

concept word, protocol supplementary concept word, rare disease supplementary concept word, unique identifier, synonyms]

2 Cognitive Behavioral Therapy/

3 Internet‐Based Intervention/

4 2 and 3

5 1 or 4

6 (‘chronic disease*‘ or ‘chronic condition*‘ or ‘chronic illness*‘).mp. [mp=title, abstract, original title, name of substance word, subject heading word, floating sub‐heading word, keyword heading word, organism supplementary concept

word, protocol supplementary concept word, rare disease supplementary concept word, unique identifier, synonyms]

7 Chronic Disease/

8 6 or 7

9 (adolescen* or teen* or youth* or juvenile* or child* or offspring*). mp. [mp=title, abstract, original title, name of substance word, subject heading word, floating sub‐heading

word, keyword heading word, organism supplementary concept

word, protocol supplementary concept word, rare disease supplementary concept word, unique identifier, synonyms]

10 8 and 9

11 5 and 10

12 (adult* or caregiver* or parent* or m?m* or dad* or mother* or father* or family* or guardian*). mp. [mp=title, abstract, original title, name of substance word, subject heading word, floating sub‐heading word, keyword heading word, organism supplementary concept word, protocol supplementary concept word, rare disease supplementary concept word, unique identifier, synonyms]

13 5 and 12

14 10 and 13

15 limit 14 to yr=1970‐December 2022

## References

[1] Edwards JD , Goodman DM. The child with severe chronic illness in the ICU: A concise review. Crit Care Med 2022;50(5):848‐859. 10.1097/CCM.0000000000005512 35234176

[2] Virella Pérez YI , Medlow S , Ho J , Steinbeck K. Mobile and Web‐Based apps that support self‐management and transition in young people with chronic illness: systematic review. J Med Internet Res 2019;21(11):e13579. 10.2196/13579 31746773 PMC6893564

[3] Fraser LK , Murtagh FE , Aldridge J , Sheldon T , Gilbody S , Hewitt C. Health of mothers of children with a life‐limiting condition: a comparative cohort study. Arch Dis Child. 2021;106(10):987‐993. 10.1136/archdischild-2020-320655 33653713 PMC8461446

[4] Aujoulat I , Dechêne S , Lahaye M. Non‐disease specific health promotion interventions for chronically ill adolescents in medical settings: A systematic review. Front Public Health 2018;6. 10.3389/fpubh.2018.00391 PMC6282057 30555811

[5] Roberts MC , Steele RG. editors. Handbook of pediatric psychology. 5th ed. Guilford Press; 2017. ProQuest Ebook Central, https://ebookcentral.proquest.com/lib/curtin/detail.action?docID=4837498

[6] Eccleston C , Fisher E , Law E , Bartlett J , Palermo TM. Psychological interventions for parents of children and adolescents with chronic illness. Cochrane Database Syst Rev. 2015;4(4):CD009660. 10.1002/14651858.CD009660.pub3 25874881 PMC4838404

[7] Cousino MK , Hazen RA. Parenting stress among caregivers of children with chronic illness: A systematic review. J Pediatr Psychol 2013;38:809‐828. 10.1093/jpepsy/jst049 23843630

[8] Emerson LM , Bögels S. A systemic approach to pediatric chronic health conditions: why we need to address parental stress. J Child Fam Stud 2017;26, 2347‐2348(9). 10.1007/s10826-017-0831-4

[9] Holly LE , Fenley AR , Kritikos TK , Merson RA , Abidin RR , Langer DA. Evidence‐base update for parenting stress measures in clinical samples J Clin Child Adolesc Psychol. 2019;48(5):685‐705. 10.1080/15374416.2019.1639515 31393178

[10] Wood BL , Miller BD , Lehman HK. Review of family relational stress and pediatric asthma: the value of biopsychosocial systemic models. Fam Process 2015;54(2):376‐389. 10.1111/famp.12139 25683472

[11] Helgeson, V. S. , Becker, D. , Escobar, O. , & Siminerio, L. (2012). Families with children with diabetes: implications of parent stress for parent and child health. J Pediatr Psychol, 37(4), 467‐478. 22267104 10.1093/jpepsy/jsr110 PMC3334535

[12] Moreland AD , McRae‐Clark A. Parenting outcomes of parenting interventions in integrated substance‐use treatment programs: A systematic review. J Subst Abuse Treat 2018;Jun 89:52‐59. 10.1016/j.jsat.2018.03.005 29706173 PMC5939592

[13] Morawska A , Calam R , Fraser J. Parenting interventions for childhood chronic illness: A review and recommendations for intervention design and delivery. J Child Health Care 2015;19(1):5‐17. 10.11771/1367493513496664 24486817

[14] Pinquart M. Parenting stress in caregivers of children with chronic physical condition—a meta‐analysis. Stress Health. 2018;34(2):197‐207. 10.1002/smi.2780 28834111

[15] Palermo TM , Putnam J , Armstrong G , Daily S. Adolescent autonomy and family functioning are associated with headache‐related disability. Clin J Pain 2007;23(5):458‐465. 10.1097/AJP.ob013e31805170e2 17515745

[16] Kim‐Cohen, J. , Moffitt, T. E. , Taylor, A. , Pawlby, S. J. , & Caspi, A. (2005). Maternal depression and children's antisocial behavior: nature and nurture effects. Arch Gen Psychiatry, 62(2), 173‐181. 15699294 10.1001/archpsyc.62.2.173

[17] Witt, W. P. , & DeLeire, T. (2009). A family perspective on population health: The case of child health and the family. WMJ, 108(5), 240‐245. 19743754 PMC2871389

[18] Golfenshtein N , Srulovici E. , Medoff‐Cooper B. Investigating parenting stress across pediatric health conditions – A systematic review. Issues Compr Pediatr Nurs 2015;14:1‐49. 10.3109/01460862.2015.1078423 26367769

[19] Law EF , Beals‐Erickson SE , Noel M , Claar R , Palermo TM. Pilot randomized controlled trial of Internet‐delivered cognitive‐behavioral treatment for pediatric headache. Headache 2015;55(10);1410‐1425. 10.1111/head.12635 26316194 PMC4715642

[20] Hayes SC , Hofmann SG. The third wave of cognitive behavioral therapy and the rise of process‐based care. World Psychiatry. 2017 Oct;16(3):245‐246. 10.1002/wps.20442 28941087 PMC5608815

[21] Hayes, S. C. (2004). Acceptance and commitment therapy, relational frame theory, and the third wave of behavioral and cognitive therapies. Behav Ther, 35(4), 639‐665. 10.1016/j.beth.2016.11.006 27993338

[22] Kabat‐Zinn, J. (2003). Mindfulness‐based interventions in context: past, present, and future.

[23] Sawyer Cohen J.A. , Semple RJ. Mindful parenting: a call for research. J Child Fam Stud 2010;19(2):145‐151. 10.1007/s10826-009-9285-7

[24] Duncan LG , Coatsworth JD , Greenberg MT. A model of mindful parenting: implications for parent‐child relationships and prevention research. Clin Child Fam Psychol Rev 2009;12(3);255‐270. 10.1007/s10567-009-0046-3 19412664 PMC2730447

[25] Kahl KG , Winter L , Schweiger U. The third wave of cognitive behavioural therapies: what is new and what is effective? Curr Opin Psychiatry 2012;25(6):522‐528. 10.1097/YCO.0b013e328358e531 22992547

[26] Öst L.G. Efficacy of the third wave of behavioral therapies: A systematic review and meta‐analysis. Behav Res Ther 2008;46(3):296‐321. 10.1016/j.brat.2007.12.005 18258216

[27] Hayes SC , Strosahl KD , Wilson KG. Acceptance and commitment therapy: An experiential approach to behavior change. Guildford Press;1999. 10.1016/S1077-7229(02)80009-8

[28] Lewinsohn PM. A behavioral approach to depression. In Friedman RJ , Katz MM , editors. The psychology of depression: Contemporary theory and research. John Wiley & Sons;1974.

[29] McCullough Jr. JP . Treatment for chronic depression: Cognitive Behavioral Analysis System of Psychotherapy (CBASP). 2003;13(3‐4) p. 241. Educational Publishing Foundation. 10.1002/jclp.10176 12858425

[30] Gilbert P. Introducing compassion focused therapy. Advances in Psychiatric Treatment. 2009;15(3):199‐208. 10.1192/apt.bp.107.005264

[31] Kohlenberg RJ , Tsai M. Functional analytic psychotherapy: Creating intense and curative therapeutic relationships. Plenum Press; 1991. 10.1007/978-0-387-70855-3

[32] Kabat‐Zinn J. Full catastrophe living: Using the wisdom of your body and mind to face stress, pain and illness. Delacorte;1990.

[33] Linehan MM. Skills training manual for treating borderline personality disorder. Guilford Press;1993.

[34] Parent J , McKee LG , N. Rough J , Forehand R. The association of parent mindfulness with parenting and youth psychopathology across three developmental stages. J Abnorm Child Psychol 2016;44(1):191‐202. 10.1007/s10802-015-9978-x 25633828 PMC4520790

[35] Campbell K , Thoburn J W , Leonard HD. The mediating effects of stress on the relationship between mindfulness and parental responsiveness. Couple Fam Psychol: Res Pract. 2017;6(1):48‐59. 10.1037/cfp0000075

[36] Zhang D , Chan SKC , Lo HHM , et al. Mindfulness‐based intervention for Chinese children with ADHD and their parents: A pilot mixed‐method study. Mindfulness. 2017;8:859‐872. 10.1007/s12671-016-0660-3

[37] Jones L , Gold E , Totsika V , et al. A mindfulness prent wellbeing course: evaluation of outcomes for parents of children with autism and related disabilities recruited through special schools. Eur J Spec Needs Educ. 2018;33:16‐30. 10.1080/08856257.2017.1297571

[38] Gouveia MJ , Carona C , Canavarro MC , Moreira H. Self‐compassion and dispositional mindfulness are associated with parenting styles and parenting stress: the mediating role of mindful parenting. Mindfulness. 2016;7(3):700‐712. 10.1007/s12671-016-0507-y

[39] Neff KD , Germer CK. A pilot study and randomized controlled trial of the mindful self‐compassion program. J Clin Psychol 2013;69(1):28‐44. 10.1002/jclp.21923 23070875

[40] Jefferson FA , Shires A , McAloon J. Parenting self‐compassion: A systematic review and meta‐analysis. Mindfulness. 2020;11:2067‐2088. 10.1007/s12671-020-01401-x

[41] Moreira H , Carona C , Silva N , Nunes J , Canavarro MC. Exploring the link between maternal attachment‐related anxiety and avoidance and mindful parenting: The mediating role of self‐compassion. Psychol Psychother: Theory Res Pract. 2016;89(4):369‐384. 10.1111/papt/12082 26493983

[42] Brandon P. Time away from “smelling the roses”: where do mothers raising children with disabilities find the time to work? Soc Sci Med. 2007;65(4):667‐679. 10.1016/j.socscimed.2007.04.007 17490797

[43] Lappalainen P , Pakkala I , Strömmer J , Sairanen E , Kaipainen K , Lappalainen R. Supporting parents of children with chronic conditions: A randomized controlled trial of web‐based and self‐help ACT interventions. Internet Interven. 2021 Mar 16;24:100382. 10.1016/j.invent.2021.100382 PMC8010620 33816128

[44] Enebrink, P , Högström J , Forster M , Ghaderi A. Internet‐based parent management training: A randomized controlled study. Behav Res Ther 2012;50(4):240‐249. 10.1016/j.brat.2012.01.006 22398153

[45] Wetterborg D , Enebrink P , Lönn Rhodin, K. et al. A pilot randomized controlled trial of Internet‐delivered parent training for parents of teenagers. J Fam Psychol. 2019;33(7):764‐774. 10.1037/fam0000541 31204818

[46] Braun L , Titzler I , Ebert, DD , et al. Clinical and cost‐effectiveness of guided Internet‐based interventions in the indicated prevention of depression in Green professions (PROD‐A): study protocol of a 36‐month follow‐up pragmatic randomized controlled trial. BMC Psychiatry. 2019;19:278. 10.1186/s12888-019-2244-y 31500602 PMC6734248

[47] Yap MB H , Mahtani S , Rapee RM , et al. A tailored web‐based intervention to improve parenting risk and protective factors for adolescent depression and anxiety problems: Post intervention findings from a randomized controlled trial. J Med Internet Res 2018;20(1):e17. 10.2196/jmir.9139 29351895 PMC5797292

[48] Spencer L , Potterton R , Allen K , Musiat P , Schmidt U. Internet‐based interventions for carers of individuals with psychiatric disorders, neurological disorders, or brain injuries: systematic review. J Med Internet Res, 2019;21(7):e10876. 10.2196/10876 31290399 PMC6647754

[49] Law E , Fisher E , Eccleston C , Palermo TM. Psychological interventions for parents of children and adolescents with chronic illness. Cochrane Database of Syst Rev. 2019;3:CD009660. 10.1002/14651858.CD009660.pub4 30883665 PMC6450193

[50] Bonnert M , Olén O , Lalouni M , et al. Internet‐delivered cognitive behavior therapy for adolescents with irritable bowel syndrome: a randomized controlled trial. Am J Gastroenterol. 2017;112:152‐162. 10.1038/ajg.2016.503 27845338

[51] Palermo TM , Wilson AC , Peters M , Lewandowski A , Somhegyi H. Randomized controlled trial of an Internet delivered family cognitive behavioral therapy intervention for children and adolescents with chronic pain. Pain 2009;146(1‐2):205‐213. 10.1016/j.pain.2009.07.034 19695776 PMC2760656

[52] Palermo TM , Law EF , Fales J , Bromberg MH , Jessen‐Fiddick T , Tai G. Internet‐delivered cognitive‐behavioral treatment for adolescents with chronic pain and their parents: a randomized controlled multi‐center trial. Pain. 2016;157(1):174‐185. 10.1097/j.pain.0000000000000348 26335910 PMC4852469

[53] Wade SL , Cassedy AE , Shultz EL , et al. Randomized clinical trial of online parent training for behavior problems after early brain injury. J Am Acad Child Adolesc Psychiatry. 2017;56(11):930‐939.e2. 10.1016/j.jaac.2017.09.413. 29096775

[54] Wade SL , Carey J , Wolfe CR. An online family intervention to reduce parental distress following pediatric brain injury. J Consult Clin Psychol. 2006a;74(3):445‐454. 16822102 10.1037/0022-006X.74.3.445

[55] Wade SL , Taylor H.G. , Cassedy A , et al. Long‐term behavioral outcomes after a randomized, clinical trial of counselor‐assisted problem solving for adolescents with complicated mild‐to‐severe traumatic brain injury. J Neurotrauma. 2015;32:967‐975. 10.1089/neu.2014.3684 25738891 PMC4492777

[56] Ellis DA , Carcone AI , Ondersma SJ , Naar‐King S , Dekelbab B , Moltz K. Brief computer‐delivered intervention to increase parental monitoring in families of African American adolescents with type 1 diabetes: a randomized controlled trial. Telemed J E Health. 2017a;23(6):493‐502. 10.1089/tmj.2016.01 28061319 PMC5488253

[57] Flujas‐Contreras J.M. , García‐Palacios A , & Gómez I. Technology‐based parenting interventions for children's physical and psychological health: A systematic review and meta‐analysis. Psychol Med 2019;49(11):1787‐1798. 10.1017/S0033291719000692 30977462

[58] Hansen A , Broomfield G , Yap MBH. A systematic review of technology‐assisted parenting programs for mental health problems in youth aged 0‐18 years: Applicability to underserved Australian communities. Aust J Psychol 2019;71(4):433‐462. 10.1111/aipy.12250

[59] Higgins J.PT , Thomas J , Chandler J , et al. Cochrane handbook of systematic review of interventions, 2nd ed. John Wiley & Sons;2019. 10.1002/9781119536604

[60] Shamseer L , Moher D , Clarke M , et al. Preferred reporting items for systematic review and meta‐analysis protocols (PRISMA‐P) 2015: elaboration and explanation. BMJ. 2015;349:g7647. 10.1136/bmj.g7647 25555855

[61] Hunot V , Moore THC , Caldwell DM , et al. ‘Third wave’ cognitive and behavioural therapies versus other psychological therapies for depression. Cochrane Database Syst Rev. 2013;(10):CD008704. 10.1002/14651858/CD008704.pub2 24142844 PMC12107518

